# Imagining interceptions: Eye movements as an online indicator of covert motor processes during motor imagery

**DOI:** 10.3389/fnins.2022.940772

**Published:** 2022-07-29

**Authors:** Alessio D’Aquino, Cornelia Frank, John Elvis Hagan, Thomas Schack

**Affiliations:** ^1^Faculty of Psychology and Sports Science, Neurocognition and Action Biomechanics Group, Bielefeld University, Bielefeld, Germany; ^2^Center for Cognitive Interaction Technology (CITEC), Bielefeld University, Bielefeld, Germany; ^3^Institute for Sport and Movement Science, Osnabrück University, Osnabrück, Germany; ^4^Research Institute for Cognition and Robotics (CoR-Lab), Bielefeld University, Bielefeld, Germany

**Keywords:** interception, motor imagery, eye movements, visual perception, covert motor processes, visuomotor system, action simulation

## Abstract

The analysis of eye movements during motor imagery has been used to understand the influence of covert motor processes on visual-perceptual activity. There is evidence showing that gaze metrics seem to be affected by motor planning often dependent on the spatial and temporal characteristics of a task. However, previous research has focused on simulated actions toward static targets with limited empirical evidence of how eye movements change in more dynamic environments. The study examined the characteristics of eye movements during motor imagery for an interception task. Twenty-four participants were asked to track a moving target over a computer display and either mentally simulate an interception or rest. The results showed that smooth pursuit variables, such as duration and gain, were lower during motor imagery when compared to passive observation. These findings indicate that motor plans integrate visual-perceptual information based on task demands and that eye movements during imagery reflect such constraint.

## Introduction

By referring to the expression “the Mind’s Eye,” we denote the capability to mentally visualize a scene or an action that do not persist in the physical world but only in our mind. It has been established, however, that the mental rehearsal of motor actions partially integrates visual-perceptual activity akin to motor actions in the form of eye movements. Eye movements not only represent a tool to study visual perception but also constitute a behavioral real-time marker for the investigation of cognitive processes linked to mentally simulated actions.

Embodied theories of the mind envision perception and action as being part of a continuum and are not considered as independent mental processes (Gibson, 1979; Prinz, 1984; Milner and Goodale, 2008). To this account, Prinz (1997) proposed a framework, known as common-coding theory, based on the assumption that perception and action do not pertain to different mental processes but both are coded in similar representational domains. Within the common-coding framework, event and action codes share a common representational content so that performing an action invokes associated perceptual events and perceiving stimuli activate associated action events. Those internal representations are described as internal models of action goals which are represented at a higher level of cognitive functioning (Jeannerod, 1994a). As a result, actions are planned and controlled in terms of their effects and depend directly from neural processes which comprise both perceptual and motor aspects (Haggard, 2005). A specific action should then access idiosyncratic perceptual representations accompanied by a distinct perceptual activation for the same action (Knoblich and Flach, 2001).

The linkage between perception and action does not only limit to motor actions but also to intended actions (Decety and Ingvar, 1990; Jeannerod, 2001). This is the case with motor simulation where mentally rehearsing an action relies on similar perceptual processes to motor actions (Grèzes and Decety, 2001; Lotze and Halsband, 2006; Holmes and Calmels, 2008). An account for the tight coupling between perception and action during motor simulation has been formulated in Hesslow’s (2002; 2012) simulation theory of cognitive function. The author proposed that associative neural processes and mechanisms are preserved between perception and action during both action execution and simulation. For instance, perceptual activity generated by a specific action can be elicited if the action was physically executed or only simulated mentally. In addition, the theory states that the behavioral and perceptual effects of simulated actions can be anticipated similarly as if the action was executed. In summary, the existence of associative neural mechanisms between perception and action allows perceptual activity in the brain to be elicited during motor simulation. Hesslow (2012) claimed that “Imagining and recalling things seen, heard or felt are essentially the same kind of processes as actually seeing, hearing or feeling something” (p. 72). This study focused on a specific type of motor simulation named motor imagery, defined as the mental state during which the representation of a given motor act, including its goals and action plans (Jeannerod, 1994b,^2001^), is internally rehearsed in memory without any overt motor output (Decety, 1996). Motor imagery also draws upon the cognitive mechanism of motor simulation and can be described in terms of its association to perceptual and motor processes.

Visual-perceptual activity during motor imagery can be quantified by measuring eye movements (Gueugneau et al., 2008; Heremans et al., 2008, 2009, 2011; Debarnot et al., 2011; McCormick et al., 2013). In a series of experiments, researchers established that certain gaze metrics, such as the location or duration of fixations, during an imagined interaction with a target, can tell us more about covert motor processes during motor imagery. For example, Heremans et al. (2008) compared the amplitude and frequency of eye movements during a cyclical aiming task in which participants were instructed to imagine a hand movement or rest while attending to the visual stimuli. Heremans and associates found that participants made less eye movements during rest and that the amplitude of saccades during imagery or rest was not affected by manipulations of inter-target distance. On the contrary, more frequent and task-related eye movements were found during motor imagery with their number and amplitude closely resembling those made during action execution. In another experiment, McCormick et al. (2013) compared eye movements during imagery and rest for a forward reach and point Fitts’ Task. As in Heremans et al. (2008), McCormick and colleagues also found that the number of fixations toward the target was significantly different between motor imagery and rest with the latter exhibiting a lower frequency of fixations. The authors proposed that the reason for differences in gaze metrics between imagery and rest arose due to the influence of covert motor processes besides purely perceptual ones. They also argued that eye movements in both studies reflected one’s capability to generate crude motor plans during motor imagery as opposed to attending the visual stimuli without being instructed to act. In a follow-up review, Causer et al. (2013) cautioned that eye movements occurring due to motor planning are likely to be dependent task characteristics and whether previous results extend to more dynamic environments are yet to be examined.

Whereas reaching toward a stationary target does not necessarily rely on continuous visual input to guide motion (Aivar et al., 2005; Hollingworth, 2012), interception requires the visuomotor system to adapt to external sources of spatial and temporal variability (Tresilian, 2005; Merchant and Georgopoulos, 2006; Sarlegna and Mutha, 2015). Based on this premise, smooth pursuit eye movements support the visuomotor system by monitoring the target motion direction, speed and contribute to the estimation of its future position (Tresilian, 2005; Zago and Lacquaniti, 2005; Brenner and Smeets, 2009, 2011). Also, smooth pursuit eye movements act as an online indicator of covert motor processes, such as motor planning, and integrate sensory information to form predictions of the target motion thus guiding manual interception (Mrotek and Soechting, 2007; Orban de Xivry and Lefèvre, 2007; Bennett et al., 2010; Brenner and Smeets, 2011; Spering et al., 2011; Fooken et al., 2016).

Additionally, if covert motor processes are elicited during motor imagery, then changes in smooth pursuit characteristics would reflect the pickup and integration of visual-perceptual information from the target to plan the interception. The rationale of the present study was to examine eye movements during imagery and rest for an interception task. Specifically, we examined the duration and gain of smooth pursuit eye movements in two experimental conditions: Motor imagery (MI) and Passive Observation (PO). It was hypothesized that smooth pursuit duration will be longer during MI than PO. Smooth pursuit duration was used to quantify the degree of continuous foveation of the target which is associated with trajectory prediction as well as interception planning and control (Wilmut et al., 2006; Brenner and Smeets, 2011). Besides, smooth pursuit gain was hypothesized to be lower during MI than PO. Smooth pursuit gain is an indicator of overall pursuit quality and is influenced by cognitive processing during motor planning (Fooken et al., 2021). Specifically, pursuit gain tends to decrease as corrective mechanisms (i.e., catch-up saccades) that reposition the eyes close to the target are suppressed (e.g., Mrotek and Soechting, 2007).

## Materials and methods

### Participants information

Twenty-four right-handed students (age: 26.9 ± 2.8, 11 females) with normal or corrected-to-normal vision, no self-declared pre-existing neurological condition, and no prior experience in the task took part in the study. The experimental procedure and written consent form for this study adhered to the ethical standards of the Declaration of Helsinki and were approved by the ethics committee at Bielefeld University. All participants gave their written informed consent to participate in the study.

### Apparatus and visual stimuli

Participants were seated approximately 70 cm in front of the computer display with keyboard and mouse adjusted at arm’s length on a 70 cm-high desk. The lower edge of the monitor was adjusted at 12 cm over the desk surface. Keyboard response (i.e., spacebar) was used to validate and transition through experimental instructions and drift correction. Performance during the trials was collected via mouse response (i.e., left click). Experimental instructions and trials used white foreground on a black background. Gaze position on the computer display was captured using the Eyelink II system (SR research, Ltd., Mississauga, Ontario, Canada). Eyelink II is a binocular head mounted video-based eye-tracker with a reported accuracy of < 0.5° and 0.01° of spatial resolution (dark pupil). Pupil position was recorded with a sampling rate of 500 Hz for each eye. As external devices, a wired keyboard (DELL L100), a USB 3-button optical wheel mouse (DELL) and a 60 Hz WFP monitor (DELL 2208) with a native resolution of 1,680 × 1,050 px were employed for this experiment. Eye movements were calibrated at the beginning and halfway through the experiment following the standard 9-points calibration provided by the manufacturers. Accuracy and precision of the calibration were assessed through a validation procedure (worst point error < 1.5°, avg error < 1.0°). The experiment was run using the open-source experiment builder OpenSesame (Mathôt et al., 2012). The code for the experiment was developed in Python (v. 2.7.8) with Psycho chosen as a back-end for the OpenSesame script. As visual stimuli for this experiment, a white ellipse (*r* = 15 px, 0.37° h, 0.39° v) superimposed over a black background was presented as the target for interception. The target was generated on the left edge of the display at either *x* = 0, *y* = 350 or *x* = 0, *y* = 700 and moved with constant speed toward the right edge of the display at *x* = 1,050, *y* = 350 or *x* = 1,050, *y* = 700, respectively. The choice of target starting position was randomized and counterbalanced for each experimental block.

### Procedure

At the beginning of the experiment, participants were requested to provide demographic information and to complete a modified version of the Edinburgh Handedness Inventory (Oldfield, 1971) and the Motor Imagery Questionnaire-Revised (MIQ-RS; Gregg et al., 2007; see section “Manipulation checks”). The task was performed under two conditions: Motor imagery (MI) and Passive Observation (PO). In the MI condition, participants were instructed to track the moving target with the eyes and to mentally simulate the interception as rapidly and as accurately as possible. Participants were asked to simulate the interception as accurately and vividly as possible by imagining the displacement of their own hand and the mouse cursor toward the target. While mentally simulating, participants were requested to keep the right hand on the mouse without performing any physical movement. All subjects were instructed to focus on both visual (i.e., seeing) as well as kinaesthetic (i.e., feeling) aspects of the interceptive movement from a first-person perspective. When ready to intercept, participants were instructed to physically trigger the left mouse button to end the trial. Imagery was performed with the eyes open to allow for gaze tracking. For the PO condition, participants were solely requested to track the target with their eyes and were instructed to rest while keeping the hand on the mouse. Each trial ended when the target reached the opposite end of the computer display with no further action required. The speed of the target was set at 30.4°/s for all trials in the experiment. A target speed of 21.9°/s was used for familiarization trials. A single trial consisted of two preliminary steps. First, participants were asked to perform a drift correction by looking at a fixation cross at the center of the computer display and press the spacebar to confirm gaze alignment. Second, subjects were instructed to move the mouse cursor to a white box in the middle of the screen (17 × 21 px). After completing this last step, participants were able to commence the trial by pressing the spacebar. At the beginning of each experimental trial, participants were asked to maintain their gaze on a fixation cross located at the center of the display. After 2000 ms, the fixation cross disappeared and the moving target appeared on the left edge of the computer display. Each participant performed a sequence of 2 blocks of 20 trials in each condition (i.e., MI and PO) counterbalanced between subjects. An average trial consisted of an initial first saccadic movement positioning the eyes in the vicinity of the target followed by a tracking phase comprising of both pursuit and small saccadic events until the time of interception. Written instructions were provided on the display at the beginning of each experimental block.

### Gaze analysis

For the purpose of the experiment, the following smooth pursuit metrics were identified.

#### Smooth pursuit duration

The duration of smooth pursuit was calculated by removing saccadic events and considering the number of samples in the interval between the first saccade and the end of a trial.

#### Smooth pursuit gain

Smooth pursuit gain was computed as the ratio between eye and target velocity. A smooth pursuit gain score closer to 1 indicated that the eyes were accurately tracking the target during the overall pursuit of the target while gain scores smaller than 1 indicated that the eyes were “lagging” behind the target.

As smooth pursuit and saccades are considered to be interdependent (Orban de Xivry and Lefèvre, 2007; Goettker and Gegenfurtner, 2021), we also analyzed the following variables to control for the effect of saccades over smooth pursuit metrics:

#### Saccadic amplitude

Saccadic amplitude has been operationalized as the Euclidean distance (expressed in ° of visual angle) between the starting and ending position of a saccade. Following previous literature (Cesqui et al., 2015), two distinct variables were analyzed: (1) the average amplitude of saccades excluding the first saccadic event, and (2) the amplitude of the first saccade toward the target.

#### Latency of the first saccade

Latency of the first saccade has been defined as the time difference (in ms) between the start of the trial and the onset of the first saccadic event.

**FIGURE 1:**
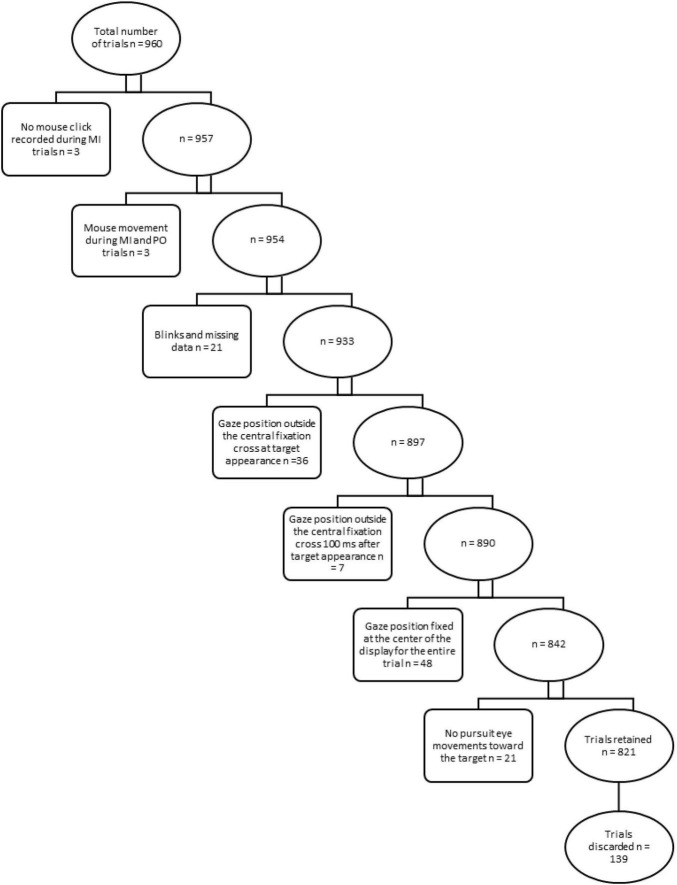
Exclusion criteria for trials. Rectangular boxes describe exclusion criteria and specify the relative number of trials discarded from further analysis in a step-wise approach. Ellipsoidal boxes indicate the relative number of trials retained after each step.

Data were pre-screened and exclusion criteria were applied to discard trials from further analyses (see Figure 1).

For the pre-screening of the data, we used SR Research Data Viewer (v. 2.4.1). Data were manipulated and analyzed using R-studio (v. 1.0.44). Next, we used a modified version of the adaptive algorithm developed by Larsson et al. (2013) for the detection and classification of saccadic events in the presence of smooth pursuit. In a first *pre-processing stage*, the averaged position coordinates for *x* and *y* were converted from px to degrees of visual angles. In a second step, artifacts (i.e., 1-sample spikes) in the eye-tracking position signal were suppressed using a 3-points running median of neighboring samples. Subsequently, a saccadic detection stage aimed at identifying approximate saccadic intervals and determining the onset and offset of saccadic events. In this step, *x* and *y* angular velocities and accelerations were, respectively, computed using a 9-points filtering approach. Then, approximate saccadic intervals were identified from the acceleration signal by identifying individual time samples exceeding a threshold of η = λσ for the *x* or *y* acceleration components – where η represents the threshold value and λ represents a constant multiplied to the standard deviation σ for the individual *x* and *y* components for each trial. In our study, we calculated σ from the acceleration interval comprised between an initial peak acceleration threshold of ± 3,000 (°/sec^2^) following a similar approach described in other studies (Nyström and Holmqvist, 2010). This procedure was necessary to prevent large spikes in the acceleration profile, usually generated by the first saccade to affect the computation of σ for the overall *x* and *y* acceleration signals. After the identification of approximate saccadic intervals, the exact and offset and onset of saccades were determined when three deviation criteria based on the physiological characteristic of saccadic eye movements were met. Contrarily to Larsson et al. (2013), we adopted a more conservative approach by classifying saccades where all the three criteria described in previous research article were met, rather than only one, to reduce the frequency of events’ misclassifications from the algorithm. For this experiment, we did not deem necessary to discriminate extremely slow or static eye movements (e.g., fixations) because they were treated as part of smooth pursuit events in accordance with other experiments (Mrotek and Soechting, 2007; Mrotek, 2013; Cesqui et al., 2015). After the implementation of the algorithm (see Table 1), misclassifications were manually corrected while saccades were filtered out of the eye-tracking signal and were analyzed separately. Following the classification procedure, trials in the PO condition were time-trimmed to the mean of MI trials for each participant. This procedure allowed to eliminate noise and irrelevant data from eye movements during PO whilst ensuring comparability at statistical level.

**TABLE 1:** Algorithm parameters for saccadic detection.

Parameter	Value	Description
**Preprocessing**		
α_min_	0.3°	Min. amplitude for a 1-sample spike
**Saccade detection**		
*t*_min_	20 ms	Min. time between two saccades
T	6 ms	Min. saccade duration
λ	6	No. of SD from the mean for the detection of saccadic intervals
δ	70°	Max. allowable deviation from the main direction
*t*_*K*_	6 ms	Max. duration of deviation from the main direction
β	40°	Largest allowable change in intra-saccadic direction
*t*_*N*_	5 ms	Max. duration of inconsistent sample-to-sample direction
M	1	No. of distance below v

### Manipulation checks

At each stage during the experiment, we conducted manipulation checks to evaluate participants’ motor imagery ability, experience, and degree of handedness.

#### Motor imagery ability

For the individual assessment of motor imagery ability, we used the MIQ-RS (Gregg et al., 2007) is designed to assess an individual’s ability to mentally “see” or “feel” simple motor actions such as movements of the arms and hands. The MIQ-RS showed acceptable internal reliability of α = 0.87 and α = 0.90, respectively, for the visual and kinaesthetic subscales as well as test-retest reliability of *r* = 0.83 for the visual subscale and *r* = 0.73 for the kinaesthetic subscale. The MIQ-RS is composed of a total of 14 items requiring a participant to predominantly perform arm and hand movements and subsequently mentally rehearse the same action. While 7 items assess the ability to “see” the imagined movement (i.e., visual imagery scale), the other 7 measure the ability to “feel” the imagined movement (i.e., kinaesthetic imagery scale). The ease or difficulty of performing each mental task is then rated on a 7-point Likert scale with a score of 1 representing “very hard to see/feel” and a score of 7 representing “very easy to see/feel.” Therefore, possible scores range from 14 (=extremely poor imager) to 98 (=extremely good imager) for the combined modalities. A cut-off score of 28 for each imagery subscale was chosen to distinguish ‘poor’ from ‘good’ imagers as suggested by the motor imagery literature investigating visual-perceptual motor tasks (Smith and Collins, 2004; Wakefield and Smith, 2009).

#### Motor imagery experience

Compliance with the experimental instructions and the overall motor imagery experience was assessed through manipulation checks as suggested by Goginsky and Collins (1996). To this regard, participants were asked to fill a short pen-and-paper questionnaire after the completion of each block of trials asking how often participants mentally imagined or physically performed the aiming movement with the mouse. For the two questions, responses were provided on a 7-points Likert Scale (1 = *Never*, 7 = *Always*). For evaluating the motor imagery experience, participants filled an evaluation form after the completion of the experiment. In this case, 8 questions evaluated on a 7-point Likert Scale (1 = *Very Easy*, 7 = *Very Difficult*) assessed the easiness to perform: as accurately and as rapidly as possible, from a first-person perspective, as clearly and as vividly as possible, in both the kinaesthetic and visual imagery modalities. Similarly, the frequency of performing from a first or third person perspectives was also assessed on a 7-point Likert Scale (1 = *Never*, 7 = *Always*).

#### Handedness

Participants’ handedness was assessed using a modified version of the original Edinburgh Handedness Inventory (Oldfield, 1971) adjusted to best fit the experimental format. The two main changes from the Edinburgh Handedness Inventory denote the exclusion of three of the original activities, the inclusion of a new one, (i.e., computer mouse), and the adoption of a 5-cells-Likert-Scale response grid.

### Statistical analyses

A Wilcoxon Signed Rank Test was used to measure the differences in gaze metrics between Motor imagery (MI) and Passive Observation (PO). Statistical significance was pegged at a *p*-value of 0.05 for screening purposes for refusing to accept or reject a hypothesis. Given that statistical significance only tells us the likelihood of an association being true, we calculated effect size using the Cohen’s d interpretation based on the magnitude of the effect size 0.1 = small, 0.3 = medium, and 0.5 = large (Cohen, 1988).

## Results

**FIGURE 2:**
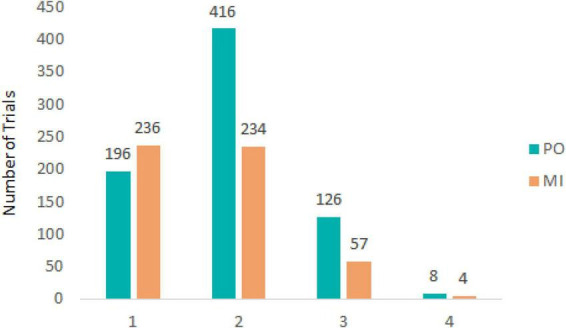
Number of trials containing a total of 1, 2, 3, or 4 saccades for PO (blue) and MI (orange).

**FIGURE 3:**
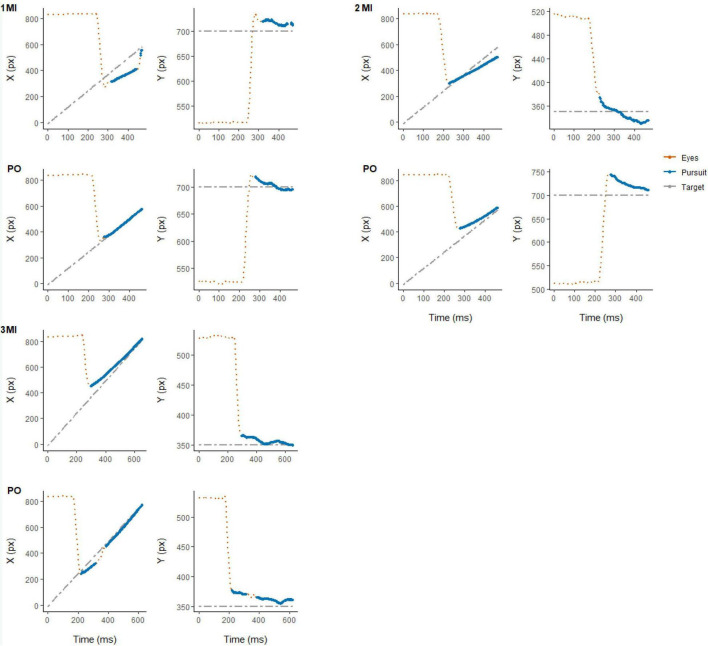
Representative example of eye (in red) and target (in gray) position for individual trials in each experimental condition (MI and PO) from randomly selected participants: 3 (top-left), 7 (top-right), and 23 (bottom-left). Eye movement signals classified as smooth pursuit are highlighted (in blue).

For both experimental conditions, each trial started with a first saccade approximately 200 ms after the onset of target motion and the disappearance of the fixation cross in the middle of the display. The saccade rapidly moved the eyes toward the target to the left side of the screen after which pursuit was initiated. After the first saccade, pursuit in most trials was characterized by no additional (432 trials, 33.8% of total) or 1 saccade (650 trials, 50.9% of total) being performed until the time of interception (see Figure 2). Examples of eye movement patterns in relation to target position for both experimental conditions are shown in Figure 3.

### Smooth pursuit duration

**FIGURE 4:**
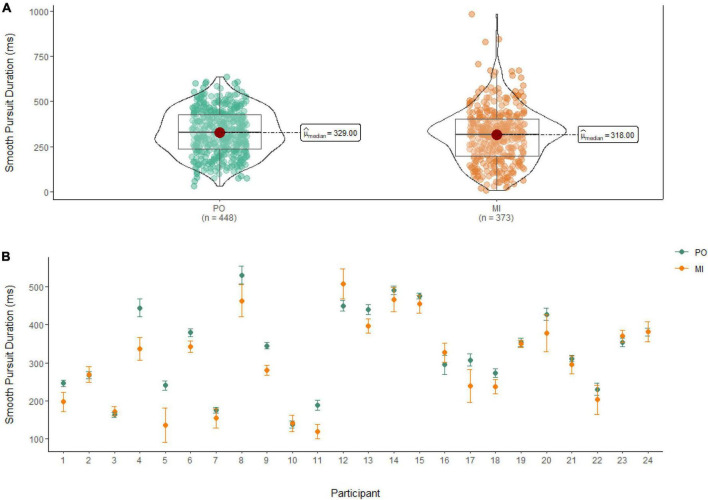
**(A)** Smooth pursuit duration differences between PO (left) and MI (right). **(B)** The average duration of smooth pursuit for PO (blue) and MI (orange) is displayed for each of the 24 participants enrolled in the experiment. Error bars are standard error of the mean.

A Wilcoxon Signed Rank Test revealed that there was a statistically significant difference in smooth pursuit duration between MI and PO, W = 91,888, *n* = 821, *p* = 0.01, with a small effect size (*r* = 0.10). The median smooth pursuit duration decreased from PO (Md = 329 ms) to MI (Md = 318 ms; see Figure 4).

### Smooth pursuit gain

**FIGURE 5:**
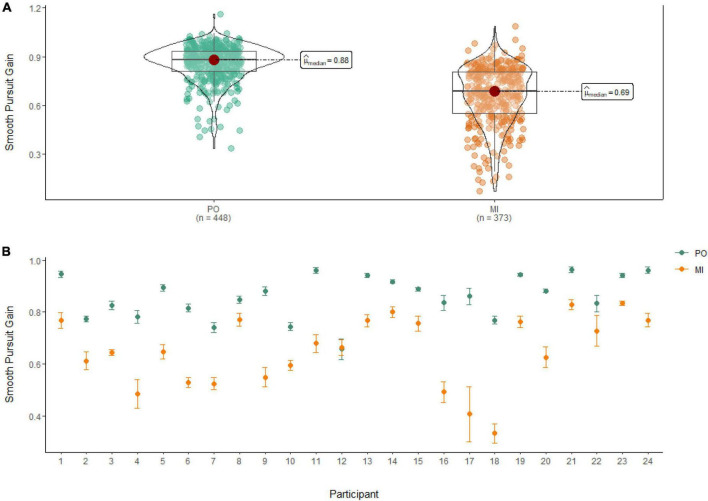
**(A)** Smooth pursuit gain differences between PO (left) and MI (right). **(B)** The average gain of smooth pursuit for PO (blue) and MI (orange) is displayed for each of the 24 participants enrolled in the experiment. Error bars are standard error of the mean.

A Wilcoxon Signed Rank Test revealed that there was a statistically significant difference in Smooth Pursuit Gain between MI and PO, W = 142,000, *n* = 821, *p* < 0.001, with a large effect size (*r* = 0.70). The median smooth pursuit gain decreased from PO (Md = 0.88) to MI (Md = 0.69; see Figure 5).

### Amplitude of saccades

**FIGURE 6:**
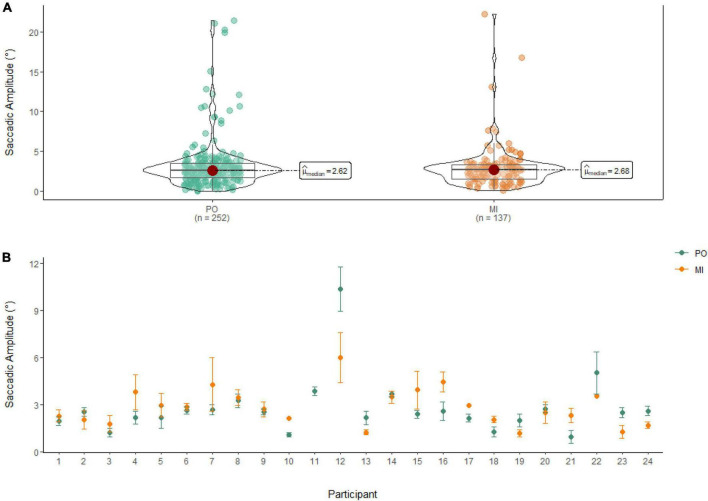
**(A)** Similarities in saccadic amplitude between PO (left) and MI (right) after the first saccade. **(B)** The average amplitude of saccades for PO (blue) and MI (orange) is displayed for each of the 24 participants enrolled in the experiment. Error bars are standard error of the mean. Missing error bars indicate that only one observation was identified. With the exclusion of the first saccade, participant 11 performed no saccades during MI.

A Wilcoxon Signed Rank Test showed that there was no statistically significant difference in the Amplitude of Saccades between MI and PO, W = 17,852, *n* = 389, *p* = 0.58. The median saccadic amplitude during PO (Md = 2.62°) was similar to the one in MI (Md = 2.68°; see Figure 6).

### Amplitude of the first saccade

**FIGURE 7:**
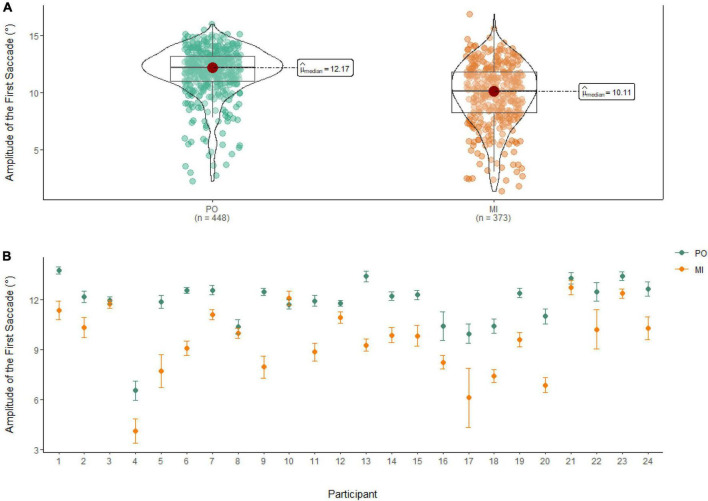
**(A)** Differences in the amplitude of the first saccade between PO (left) and MI (right). **(B)** The average amplitude of the first saccade for PO (blue) and MI (orange) is displayed for each of the 24 participants enrolled in the experiment. Error bars are standard error of the mean.

A Wilcoxon Signed Rank Test showed that there was a statistically significant difference in Amplitude of the First Saccade between MI and PO, W = 122,000, *n* = 821, *p* < 0.01, with a medium effect size (*r* = 0.46). The median amplitude of the first saccade was higher in PO (Md = 12.17°) when compared to MI (Md = 10.11°; see Figure 7).

### Latency of the first saccade

**FIGURE 8:**
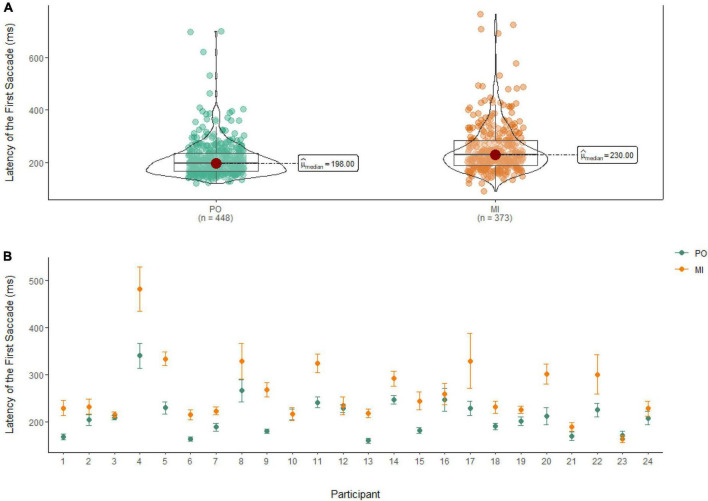
**(A)** Differences in the latency of the first saccade between PO (left) and MI (right). **(B)** The average latency of the first saccade for PO (blue) and MI (orange) is displayed for each of the 24 participants enrolled in the experiment. Error bars are standard error of the mean.

A Wilcoxon Signed Rank Test revealed that there was a statistically significant difference in the Latency of the First Saccade between MI and PO, W = 58,727, *n* = 821, *p* < 0.01, with a moderate effect size (*r* = 0.30). The median amplitude of the latency of the first saccade was lower in PO (Md = 198 ms) when compared to MI (Md = 230 ms; see Figure 8).

## Discussion

Aiming and pursuing objects under visuo-spatial guidance are key motor behaviors of everyday life (de Brouwer et al., 2021; Fooken et al., 2021). In this study, we investigated eye movements during motor imagery of an interception task and passive observation of a moving target. We found that smooth pursuit characteristics while imagining an interception tend to differ from those in which the target is passively tracked (i.e., no intention to intercept). Contrarily to our hypotheses, we found that participants track the target for shorter time and with lower gain during imagery when compared to passive observation with these effects being moderated by the time of the onset of the first saccade. These results are in line with the general idea that eye movements are influenced by covert motor processes during imagery. We also argue that smooth pursuit characteristics seem to depend on the visuomotor demands of the task.

Research has already shown that motor imagery replicates perceptual and cognitive processes of motor actions (Papaxanthis et al., 2002; Gentili et al., 2004). One goal of interceptive tasks is to predict the target’s motion so that an interception can be planned ahead of time. In the current study, the decreased duration of tracking during MI might be explained by the engagement of motor planning aimed at predicting the future state of the target. In those circumstances where the target moves linearly with a high motion predictability, as in the current experiment, tracking the target throughout its displacement could improve the temporal estimation of target motion. The relatively low average amplitude of saccades which landed in close proximity to the target combined with the infrequent presence of large saccades (i.e., amplitude > 10°), show that it is likely that those saccades were triggered by the accumulation of positional error between the eyes and the target. This result further endorses the idea that a mechanism of saccadic suppression is active during MI as a direct consequence of motor planning and that the occurrence of predictive saccades was not a general trend. In MI, participants track the target with a lower degree of accuracy (i.e., lower smooth pursuit gain than PO) and seem unable to compensate for the accumulation of positional error with larger saccades. As previously indicated, saccadic suppression may boost the temporal estimation of target motion (Goettker et al., 2019) while predictive saccades are likely to improve spatial accuracy (Fooken and Spering, 2020). It must be noted that saccadic suppression usually occurs when the trajectory of the target is unpredictable and continuous monitoring of the target is necessary to extrapolate motion parameters (Fooken et al., 2021). In our experiment, only the target initial position was manipulated while speed and trajectory were kept constant. We argue that temporal constraints might play a critical role in the modulation of the mentally simulated interception and that keeping the eyes on the target may boost temporal accuracy. During normal interceptions, it has been shown that visuomotor task demands influence the degree of temporal or spatial precision (Tresilian and Lonergan, 2002; Fooken et al., 2021). In MI, the absence of spatial information such as the relative position between the cursor and the target combined with the lack of proprioceptive information to guide and correct the hand movement might contribute to the reliance on visual information from target motion to estimate optimal interception time. This conclusion is in line with previous literature suggesting that removing visual and proprioceptive consequences of an action affects the saccadic system during error correction and shifts focus toward the estimation of temporal parameters to allow for optimal performance (Huang and Hwang, 2012). One consideration to be taken into account in the current study is that similarity in saccadic amplitude between MI and PO does not imply similarity in function. One hypothesis is that saccades during MI still retain a predictive function perhaps in dependance to interception strategies (Fooken et al., 2016). Hence, it could be inferred that smooth pursuit eye movements could be used not only as a dynamic marker for the evaluation of motor imagery capacity but also to understand motor planning during motor imagery.

The decrease in smooth pursuit duration from PO to MI could be seen as perceptual strategy that mirrors an information-processing demand required for motor task (Liversedge and Findlay, 2000; Henderson, 2003). Behavioral evidence reveals that the eyes usually reach at the target before the hand attains peak velocity, allowing adequate time for adjustment processes to occur (Starkes et al., 2002). The present finding also reiterates the eye–hand coordination perspective on the spatiotemporal connection between the eye and hand control systems during goal-directed actions (Stein and Glickstein, 1992; van Donkelaar, 1997; Helsen et al., 2000; Fooken et al., 2021). It can further be hypothesized that high-acuity vision through smooth pursuit eye movements during imagery seem to play a functional role in the generation of the motor plan. Previous literature has been established that motor imagery and execution rely on similar motor representations and neural substrates (Grèzes and Decety, 2001; Holmes et al., 2010). Those neural networks allow imagery to mimic the spatial and temporal dynamics of motor actions through the engagement of internal forward models (Flanagan and Johansson, 2003). During interceptions, perceptual activity allows to overcome sensorimotor delays generated by the object’s motion with respect to movement of the arm and hand (Fooken et al., 2021). Similarly, smooth pursuit eye movements during imagery support the visuomotor system by helping to generate accurate motion predictions which are functional when planning and simulating the interceptive movement.

In our task, participants were required to track a “fast” (≈ 30°/s) moving target and, in the imagery condition, they were additionally instructed to mentally simulate the interception as fast and as accurately as possible. Smooth pursuit eye movements significantly lagged behind the target during MI (Md = 0.69) whereas more accurate pursuit was found for PO (Md = 0.88). These results are in line with the interception literature showing that gain values tend to deteriorate when covert motor processes are involved. Mrotek and Soechting (2007) found that participants rely on smooth pursuit eye movements to continuously monitor the target and suppress corrective eye movements when moving to intercept. When saccades are suppressed, position error between the eyes and the target accumulates since the smooth pursuit system alone cannot reach high velocities and gaze tend to lag behind the target. When participants are only instructed to track the target, however, the relative position between the eyes and the target tends to keep relatively stable with higher gain values. Instead, lower values of smooth pursuit gain during MI recorded in the present work suggest that eye movements during imagery are created as action sequence in several frames under different conditions (Kosslyn et al., 1995). McCormick et al. (2013) reiterated that for different tasks and conditions, eye movement characteristics change as a function of both spatial and temporal requirements. Whereas lower gain values are found when covert motor processes are involved, smooth pursuit duration seems to be influenced by the visuomotor demands of the task. In general, accurate motion perception is crucial for interception planning (Brenner and Smeets, 2011) and participants commonly rely on smooth pursuit to maintain high-acuity vision of the target (Spering et al., 2011). While these conclusions are generally true for targets that are relatively unpredictable or are temporally occluded (Fooken et al., 2016, 2021), observers may also benefit from using prior knowledge and cognitive heuristics to simulate the interception when targets are fast and predictable. This idea supports the hypothesis that smooth pursuit characteristics are both affected by task requirements as well as internal states. Fooken and Spering (2019, 2020) demonstrated that internal decisions states during go/no-go interception task are associated with idiosyncratic smooth pursuit profiles. Specifically, decisions to intercept were associated with larger position errors between the eyes when compared to no-go decisions. In our experiment, pursuit is always initiated by single saccade redirecting gaze toward the moving target. The amplitude and size of this initial saccade is associated with object and motion discrimination as well as following smooth pursuit quality (Land and McLeod, 2000; Fooken et al., 2016). Higher delay and lower amplitude of the first saccade seem to contribute to lower smooth pursuit duration during MI. As the latency of the first saccade increases, the target moves toward the center of the screen where relatively smaller saccades are needed to relocate the fovea in its vicinity. Besides stimulus properties, a range of “top-down” cognitive processes has been shown to influence saccadic characteristics [see Hutton (2008) for a review]. One hypothesis is that image generation and maintenance interfere with the perceptual processes related to object detection. It has been shown that visual components of imagery can interfere with perception when both occur concurrently (Pearson et al., 2008; Reeves et al., 2020). Hence, engaging and maintaining the motor image while perceiving could have resulted in higher initial saccadic latency due to reduced peripheral acuity. These findings suggest that smooth pursuit characteristics during motor planning are influenced by the interaction between task characteristics and internal states.

## Limitations

A limitation of the present study is that the study of smooth pursuit characteristics provides a general overview about the involvement of motor processes during imagery but does not offer an account over the unfolding of the simulated interception task. Future studies could assess whether dynamic changes in smooth pursuit gain and duration over individual trials can provide a temporal demarcation of different stages of motor planning. On a similar note, the current study provides no direct comparison between motor imagery and physical interceptions. While smooth pursuit characteristics during imagery seems to be influence by motor planning processes similarly to execution, it might be that the two conditions take into account different factors due to simulation constraints. For example, the absence of proprioceptive or visual cues from the hand could lead to using predictive cues to simulate interception during imagery while current information from the limb displacement could guide and affect motor planning during execution. Therefore, an open question remains on whether motor imagery takes into account and integrate preliminary environmental constraints or replicate, to any degree, control strategies exhibited during normal intercept for the generation of motor plans.

## Conclusion

Our findings suggest that smooth pursuit characteristics during imagery are influenced by covert motor processes, such as motor planning. This study extends previous findings examining gaze behavior toward goal-directed actions during motor imagery by establishing eye movements as a reliable indicator to investigate covert motor processes in complex and dynamic environments.

## Data availability statement

The raw data supporting the conclusions of this article will be made available by the authors, without undue reservation.

## Ethics statement

The studies involving human participants were reviewed and approved by the study was conducted according to the guidelines of the Declaration of Helsinki, and approved by the Ethics Committee of Bielefeld University (protocol code No. 2022-096 of 25 April 2022). The patients/participants provided their written informed consent to participate in this study.

## Author contributions

AD, CF, and TS designed the experiment. AD collected and analyzed the data and wrote the manuscript. AD and JH interpreted the data. All authors reviewed the manuscript. All authors have read and agreed to the published version of the manuscript.
